# Age-Related Intrinsic Functional Connectivity Changes of Locus Coeruleus from Childhood to Older Adults

**DOI:** 10.3390/brainsci11111485

**Published:** 2021-11-10

**Authors:** Inuk Song, Joshua Neal, Tae-Ho Lee

**Affiliations:** 1 Department of Psychology, Virginia Tech, Blacksburg, VA 24060, USA; psyence221b@vt.edu (I.S.); jneal96@vt.edu (J.N.); 2 School of Neuroscience, Virginia Tech, Blacksburg, VA 24060, USA

**Keywords:** locus coeruleus, distractibility, neurodevelopment, functional connectivity

## Abstract

The locus coeruleus is critical for selective information processing by modulating the brain’s connectivity configuration. Increasingly, studies have suggested that LC controls sensory inputs at the sensory gating stage. Furthermore, accumulating evidence has shown that young children and older adults are more prone to distraction and filter out irrelevant information less efficiently, possibly due to the unoptimized LC connectivity. However, the LC connectivity pattern across the life span is not fully examined yet, hampering our ability to understand the relationship between LC development and the distractibility. In this study, we examined the intrinsic network connectivity of the LC using a public fMRI dataset with wide-range age samples. Based on LC-seed functional connectivity maps, we examined the age-related variation in the LC connectivity with a quadratic model. The analyses revealed two connectivity patterns explicitly. The sensory-related brain regions showed a positive quadratic age effect (u-shape), and the frontal regions for the cognitive control showed a negative quadratic age effect (inverted u-shape). Our results imply that such age-related distractibility is possibly due to the impaired sensory gating by the LC and the insufficient top-down controls by the frontal regions. We discuss the underlying neural mechanisms and limitations of our study.

## 1. Introduction

The locus coeruleus (LC) is a small nucleus located deep in the brainstem and a major source of norepinephrine. The LC releases norepinephrine to almost the entire brain throughout its efferent projections according to both the phasic and tonic firing of LC neurons, thereby the LC is one of the primary brain regions critical for selective information processing by changing the brain’s configurations [1,2,3,4,5,6,7,8,9,10]. Recent studies consistently have suggested that the LC functionally controls sensory inputs at the early sensory gating stage by changing the brain’s connectivity configurations [11,12,13]. For instance, the stimulated LC changes its neural communication with the basolateral nucleus of the amygdala [14] and thalamus [15,16], which receive the majority of sensory information for the further in-depth cognitive process. Similarly, direct chemogenetic stimulation of LC immediately changes neural connectivity configurations, especially for the intrinsic networks that mediate the bottom-up sensory process, including the primary sensory and salience networks [17]. That is, the LC plays a role in controlling sensory flows in the brain, suggesting that impaired processing selectivity is possibly due to the failure of communication between the LC and sensory regions that introduces the sensory overflows in the brain.

Human imaging studies also imply that the LC system changes the bottom-up process at the early sensory-perceptual stage to prioritize important information. For example, the induced phasic LC activity by arousing or stressful stimuli at the early sensory-perceptual level increases the initial selective attention processes [7,8,9] and attentional control [18,19]. Finally, such initial LC-induced selectivity at the early sensory stage carries over to the late cognitive processing such as memory encoding [20], memory consolidation [5,21], and decision making [22]. Thus, the interrupted LC activity has been often examined in individuals with conditions associated with hyperarousal and attentional vigilance such as attention-deficit hyperactivity disorder (ADHD) [23,24,25], and posttraumatic stress disorder (PTSD) [26,27,28].

Recent studies also showed that older adults, who are more prone to distraction, exhibit interrupted LC connectivity [29,30,31,32,33,34,35,36]. For instance, older adults showed the hyper-connectivity of the LC with the primary sensory networks compared to younger adults as well as the hypo-functional coupling with the salience network [8], suggesting that the impaired connectivity between the sensory regions and LC induces sensory overflows in the brain and thus the salience network fails to guide attention appropriately. As a result, there is an unnecessary depletion of limited neural resources in the brain leading the executive frontal systems to not maintain goal-directed processes due to irrelevant stimuli that should be ignored earlier. 

However, children’s developmental trajectories of LC connectivity have not been characterized yet. Healthy young children also show behavioral propensity to react to irrelevant information combined with heightened impulsivity [37,38,39]. This distractibility in children is possibly due to their less-developed LC function during their early developmental stage, such that structural studies have demonstrated that structural integrity of the LC increases with age gradually and then declines after the peak (i.e., inverted U-shaped curvilinear trend) [40,41,42]. Furthermore, studies suggest that brain development occurs first in the primary sensory bottom-up regions from early childhood with a progressively maturing top-down frontal system [43,44]. Thus, the unbalanced brain development in childhood between not-fully-developed LC and matured sensory network regions possibly leads children to fail at moderating sensory overflows in the brain.

Considering accumulating evidence indicating that the LC plays an essential role in the brain’s processing selectivity, the overarching objectives of the current study are to provide a full description of LC connectivity pattern across the lifespan from early childhood to older adulthood. Given the brain development findings [40,41,42] and heightened distractibility in early childhood and older adults [7,8,37,38,39], we hypothesized that children and older adults, compared to younger adults and middle adults, show increased functional connectivity of the LC with the primary sensory regions (i.e., quadratic or u-shape curve), indicating that those two age groups have unnecessarily higher sensory sensitivity intrinsically even without task-induced activity (i.e., resting-state fMRI). We hypothesized the quadratic age effects because the development of structural integrity in the LC follows a curvilinear trend [40] and behavioral performances also follow the inverted U-shape in general [45]. To this end, we used cross-sectional samples (age-ranged between 8 and 83 years) and examined intrinsic functional connectivity of the LC associated with age changes based on the resting-state fMRI data. We especially examined the intrinsic network connectivity of the LC based on the resting-state fMRI signal as it reflects general intrinsic neural architecture of brain development at the time of the brain scan, rather than a moment-by-moment task-specific neural response [8,46,47,48,49].

## 2. Materials and Methods

### 2.1. Data Characteristics

The present study was carried out using resting-state fMRI data from the enhanced Nathan Kline Institute (NKI)-Rockland project [50]. The dataset was initially downloaded through the Mind Research Network’s collaborative informatics and neuroimaging suite (COINS) [51]. We only included individuals with full-coverage of both T1 and EPI, and without severe motions (framewise displacement, *FD* > 0.5 mm), which resulted in 595 samples (*M* = 39.47 years, *SD* = 20.51, range = 8–83, 63.36% females; see Figure S1 in Supplementary Information). In this research, considering the previous studies [7,8,21,35], we referred to age groups approximately as follows: early childhood (<12), adolescents (12–20), younger adults (20–40), middle adults (40–60), and older adults (>60). All individual data were collected in the same scanning protocol with a 32-channel head-coil for the high-resolution structural image (T1-MPRAGE; TR = 1950 ms; TE = 2.52 ms; FA = 9°; 1-mm isotropic voxel; FOV = 256 mm) and EPI image (364 volumes; 2-mm isotropic voxel, 64 slices; TR = 1400 ms; TE = 30 ms; FA = 65°; matrix size = 112 × 112; FOV = 224 mm).

### 2.2. Preprocessing

**Figure 1:**
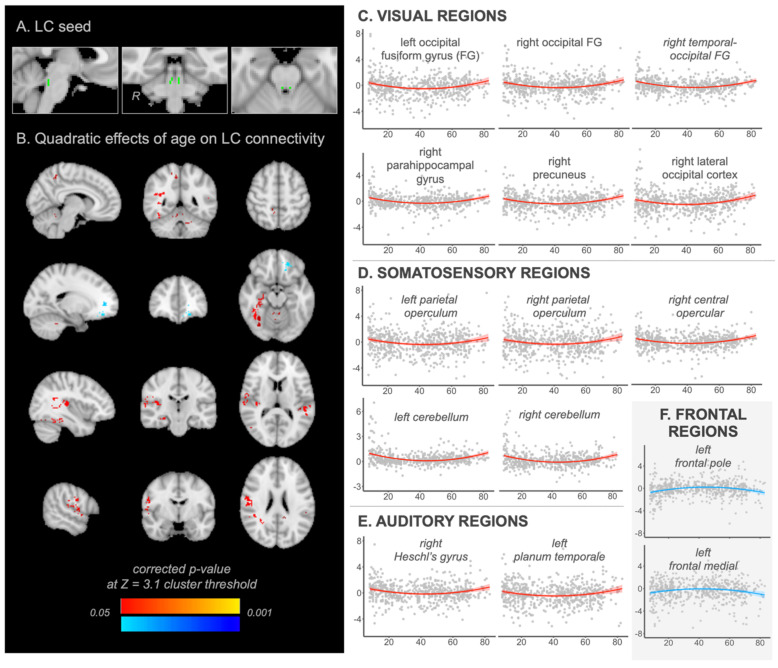
(**A**) LC seed mask (**B**) whole brain maps showing a quadratic effect of age on LC connectivity. Red: corrected *p*-value for the positive quadratic effect (u-shape curve). Blue: corrected *p*-value for the negative quadratic effect (inverted u-shape curve). Regions in (**C**) the visual network (**D**) somatosensory network (**E**) auditory network and (**F**) frontal network. *Y*-axis: LC connectivity strength with each region (z-statistics); *X*-axis: age (years).

Preprocessing was performed using the FMRIB Software Library (FSL) combined with ICA-AROMA [52] and ANTs [53], including skull stripping and tissue mask segmentation (CSF/WM/GM) after bias-field correction for structural images, and first 10-volumes cut, motion correction, slice-timing correction, intensity normalization, regressing out CSF/WM with individually segmented masks, ICA-denoising (corrected mean FD = 0.02 mm, range = 0.01–0.14 mm; Figure 1B) and registration to standard Montreal Neurological Institute (MNI) 2-mm brain template for functional images. To avoid possible signal mixture of LC region with neighboring regions such as periaqueductal gray or ventral tegmental area, we skipped the signal smoothing step. 

### 2.3. Whole-Brain Multiple Regression Analysis for Age-Related Changes in LC Connectivity

We first extracted the mean time-series of LC activity from the preprocessed image on each individual’s non-smoothed native space using a standard structural LC mask (Figure 1A) [54]. Using this LC time course, a multiple regression analysis was then performed to estimate individual level LC-seed functional connectivity maps (z-transformed). Finally, changes of LC connectivity with age were estimated at the whole-brain level using a multiple regression model. Consistent with our main hypothesis, we examined the age-related variation in the LC connectivity with a quadratic model (i.e., *age^2^* and *age*):

LC connectivity (Y) = Intercept + β_1_ (age^2^) + β_2_ (age) + β_3_ (gender) (1)

In the model, we included *gender* in the design matrix as nuisance regressors to attenuate gender effects. The group-level whole-brain connectivity model was tested using non-parametric permutation-based inference (FSL’s randomise tool with 5000 permutations) [55] with cluster threshold at *Z* = 3.1 (*p* = 0.001) and an *FWE-corrected p* at 0.05.

## 3. Results

Whole-brain multiple regression analysis on the LC connectivity revealed significant regions that have quadratic relationships with age. As expected, a significant positive quadratic relationship of age was found for connectivity between the LC and several other regions that are mainly associated with the sensory process (i.e., visual, somatosensory, auditory; Figure 1C–E). For example, visual processing regions along the ventral occipitotemporal and dorsal visual pathways including the occipital and temporal fusiform gyrus, parahippocampal gyrus, and precuneus [56], decrease functional connectivity with the LC gradually from early childhood years to a low around 40–45 years old, and then increase according to age (Figure 1C). The parietal operculum extended to the central region and the cerebellum also showed the same u-shape curve of age effects on the LC connectivity (Figure 1D). These regions are known as the secondary somatosensory cortex involved in tactile and pain sensations [57]. Finally, we found that regions in the primary auditory network including Heschl’s gyrus extended to the planum temporale [58] showed the same quadratic age relationship on the LC connectivity (Figure 1E). To sum, these results indicated that during their respective development stages, children and older adults have increased sensory interaction with the LC in the brain.

Importantly, we also found that there was a significant age-related negative quadratic effect on the LC connectivity for the frontal regions (Figure 1F). The frontal pole extended to the frontal medial cortex, known to be involved in action monitoring and cognitive control (e.g., action selection) [59], showed lower LC connectivity during the early childhood and older adulthood years than younger and middle adulthood years (i.e., inverted U-shaped curve). In other words, the LC has stronger connectivity with the frontal regions during younger and middle adulthood years compared to both developing children and older adults. All significant regions of LC connectivity associated with ages are displayed in Table 1 and Figure S2 in Supplementary Information. The group-mean of LC connectivity across ages is in Supplementary Information (Figure S3).

## 4. Discussion

The goal of the current study was to provide a full description of age-related changes in the intrinsic LC connectivity by adopting cross-sectional fMRI data from early childhood to older adulthood. Specifically, given the findings that children and older adults are prone to distraction [7,8,29,30,31,32,33,34,35,36,37,38,39], we hypothesized that the LC, a critical region for selective information processing in the brain, showed distinct connectivity patterns with other regions in early childhood and older adulthood compared to younger and middle adults who show more stable attentional ability. As a result, we found that the LC’s connectivity with sensory regions showed a U-shaped curve pattern across ages, indicating that the sensory regions exhibit highly increased intrinsic connectivity with the LC in both early childhood and late older adulthood. The current findings suggest that such age-related distractibility is possibly due to the insufficient sensory gating process by the LC. Most importantly, the current analyses also revealed that the LC connectivity with the frontal regions showed an inverted U-shaped curve pattern. That is, while the sensory network regions are connected to the LC excessively, the frontal network regions have decreased connectivity with the LC, implying that the frontal control regions cannot handle the sensory overflows appropriately. These results are similar to the previous finding in that the LC showed curvilinear connectivity patterns with other cortical regions as a function of age [41]. However, the previous study’s curvilinear patterns were cubic mostly, and most of them appeared in the frontal lobe rather than throughout the brain. In addition, our results showed two distinctive connectivity change patterns and covered a wider range of ages including early childhood. This is the first full description of how the LC configuration changes from early childhood to older adulthood, informing the LC model of distractibility in both children and older adults.

The current findings implied that the increased distractibility at both early and late developmental stages is due to not only the LC-related excessive sensory overflows but also the lower LC connectivity in the frontal regions. However, although the observed patterns of LC connectivity for sensory and frontal regions are the same for both children and older adult groups, the underlying neural mechanisms for the attentional deficits regarding the LC connectivity may not be identical given structural differences in the developmental trajectory. At the early developmental stage, the primary brain structures including sensory cortex and subcortical bottom-up network regions mature first while the higher-cognitive prefrontal regions are still in the process of developing [43,60], whereas the LC does not yet fully function [40,41,42,61]. Thus, it is possible that the LC fails to appropriately prioritize sensory inputs inflow from the fully developed sensory networks, leading to sensory overflows in the children’s brain. That is, the immature LC which cannot control sensory inputs appropriately, and the overflow leads to the unnecessarily increased functional connectivity between LC and sensory regions. In addition, the LC fails to initiate the frontal control region for the flux of sensory inputs in the children’s brain, leading to the decreased functional connectivity. In contrast, given the finding of prominent age-related decline in brain volume as well as functional response of the prefrontal areas compared to other regions [62,63], the increased distractibility in the older adults is more derived from the decreased frontal functionality in controlling sensory inputs. Although the sensory networks also showed cortical thinning with the frontal cortex in older adults [64], evidence indicates decreased sensory sensitivity in the brain at the early sensory gating stage. For instance, older adults have less activations in the visual and auditory cortex under the passive stimuli presentation [65] suggesting that older adults have less sensory-perceptual sensitivity in terms of change detection. Therefore, the LC can still handle the reduced primary sensory processing even when the LC is functionally degraded, but the prominently decreased frontal control regions are overwhelmed by even less sensory inputs. It may cause the increased functional connectivity between the LC and the sensory and the decreased connectivity between the LC and the frontal control region.

In the current study, we mainly examined the LC-centered neural connectivity across age which may possibly serve as the underlying neural mechanism for the attentional distractibility often observed at both early and late developmental stages. However, the suggested LC circuit mechanism sheds light on understanding other late cognitive process and attention-related mental disorders, as the current result showed the intrinsic connectivity pattern of the LC as a function of age, and it can be used as a framework to interpret the LC-involved neural activities. For instance, the attentional process involved in memory encoding. Some studies revealed that the LC is associated with memory encoding [5,20] and older adults with reduced LC structure showed poorer memory encoding [66]. With regard to these studies, our results imply that the intrinsic LC-parahippocampal gyrus connection is a pivotal neural circuit of memory encoding in aging. As another example, ADHD is regarded as a mental illness characterized by hyperarousal and attentional vigilance [24]. As described above, it is known that the LC is associated with ADHD. Our results may bring insight into understanding and/or predicting the neural underpinnings of ADHD developmental trajectories given that there have not been many studies involving adults with ADHD [67].

However, there are some limitations in the current study. We examined intrinsic functional connectivity of the LC using the non-task based intrinsic neural network (i.e., resting-state fMRI) based on the previous behavioral observations of the increased attentional distractibility in the early childhood and older adulthood. Thus, our observation might be suboptimal to link actual attentional ability and LC-associated neural configurations compared to task-based assessments in the laboratory with various attentional tasks, which measure attentional selectivity and control more directly. We conjecture that additional attention-related brain regions and/or networks, such as dorsolateral prefrontal cortex, anterior cingulate cortex [68,69], and frontoparietal network [70,71], can be more involved in cognitive processing and the interactions between the additional regions and the LC can be estimated. Future research is needed to employ task-based assessments to link the attentional ability and LC connectivity changes across ages. 

Moreover, it is important to note that the LC is an exceptionally small structure in the brainstem, and thus it is difficult to locate its location and signal in an individual brain. Although we used the standard LC structure mask and extracted LC time-series (i.e., LC’s neural activity) from non-smoothed EPI image on the native space [72] combined with the ICA-denoising [5,7,9] to increase LC signal fidelity in the connectivity estimation, there are several ways to increase LC signal reliability. First, an additional T1-FSE scan (i.e., structural MRI scanning for norepinephrine neuron) can be used. With a 2–3 min duration in a scan, it allows to locate the individual neuromelanin structure such as substantia nigra, ventral tegmental area, and the LC on the native space [30,73]. Recent studies also suggested that T1 structural MRI with magnetization transfer (MT-weighted MRI) can distinguish the LC from its surroundings [74,75]. Unfortunately, the current study is based on public data and thus we could not utilize additional LC structure images given the pre-determined imaging protocols and collections. Secondly, extracting a seed signal without smoothing on the native space can minimize the mixture of signals between nearby regions [8]. In our analysis, we used the non-smoothed LC time-series as a seed region neural activity. Thirdly, recent studies suggested a comprehensive mask [76] and a high-confidence meta mask of the LC by aggregating multiple LC masks [77]. For example, Dahl and colleagues yielded the high confidence mask by aggregating 6 LC masks [40,78,79,80] and showed that the meta mask captured LC-related hyperintensity accurately. Thus, it would also be beneficial to use the comprehensive or meta mask in the future study. Lastly, the LC is often confounded by physio artifacts such as cardiac pulsation and thus it is helpful to run additional physiological denoising [81]. Although the ICA-denoising is a promising approach to mitigate physiological influence at the global level, the individual-based physiological denoising process using respiration and cardiac pulse signal can be more focal and direct to the brainstem signal fluctuation correction [81]. In this instance we could not use individual specific LC masks or physiological noise correction. Therefore, in future work, it would be beneficial to utilize the LC structural scan and physiological data collection.

## 5. Conclusions

We analyzed the age-related LC connectivity changes with a quadratic model. A positive quadratic relationship of age was found for connectivity between the LC and sensory regions. A negative quadratic relationship was found between LC and frontal regions. Our results suggest that the increased distractibility at both early and late developmental stages is due to not only the LC-related excessive sensory overflow but also the lower LC connectivity with the frontal regions. It is noteworthy that the LC showed two distinctive connectivity change patterns as a function of age. Furthermore, we revealed the children’s LC connectivity configuration that has not been characterized yet. Our findings are the first full description of how the LC connectivity configuration changes across age.

## Figures and Tables

**Table 1:** Significant brain regions of quadric age effects (cluster threshold at Z = 3.1 and corrected *p*-value at 0.05 after 5000 permutation) on the LC seed-based whole-brain connectivity analysis. *t* = t-value; H = hemisphere; BA = Broadman area; Region labeling is based on Harvard–Oxford atlas.

	*t*	H	BA	MNI			*Note*
				*x*	*y*	*z*	
**Positive quadratic age effect**							
Parahippocampal gyrus	4.79	R	36	30	−22	−16	Visual
Precuneus	4.71	R	7	8	−48	52	Visual
Lateral occipital gyrus	4.37	R	19	46	−66	18	Visual
Fusiform gyrus, temporal occipital	4.52	R	37	40	−48	−22	Visual
Fusiform gyrus, occipital	4.16	L	19	−28	−78	−12	Visual
	4.07	R	37	34	−70	−16	Visual
Cerebellum	4.58	R	-	4	−56	−18	Somatosensory
	4.17	L	-	−4	−48	−16	Somatosensory
Opercular cortex, central	4.25	R	6	54	−2	6	Somatosensory
Operculum, parietal	3.81	L	13	−46	−34	20	Somatosensory
	3.27	R	13	42	−26	18	Somatosensory
Heschl’s Gyrus	4.54	R	41	38	−24	12	Auditory
Planum temporale	3.33	L	41	−42	−34	12	Auditory
**Negative quadratic age effect**							
Frontal pole	3.897	L	10	−28	52	2	Frontal
Frontal medial cortex	3.160	L	11	−4	40	−18	Frontal

## Data Availability

The enhanced NKI data are available in COINS (https://coins.trendscenter.org/) and the NITRC (https://fcon_1000.projects.nitrc.org/indi/pro/nki.html).

## Supplementary Materials

The following are available online at https://www.mdpi.com/article/10.3390/brainsci11111485/s1, Figure S1: Age distribution.; Figure S2: The whole-brain map of LC seed-based functional connectivity showing the quadratic effects of age.; Figure S3: Group-level mean LC connectivity without age consideration.
