# Supplementary material for: Age-Related Intrinsic Functional Connectivity Changes of Locus Coeruleus from Childhood to Older Adults
Source: Brain Sci. 2021 Nov 10;11(11):1485. doi: 10.3390/brainsci11111485 (PMC8615904; doi:10.3390/brainsci11111485)
Supplement: Supplementary file 1 [file brainsci-11-01485-s001.zip › brainsci-1433166-supplementary.pdf]

# Supplementary Information

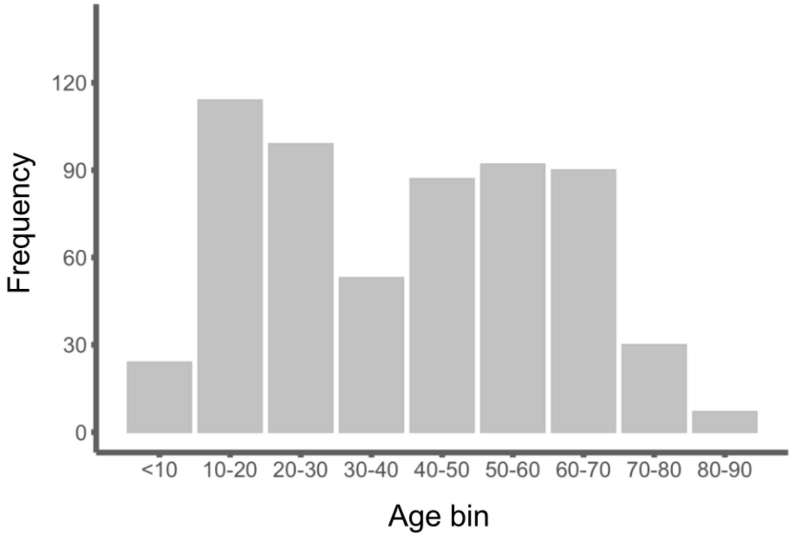


**Figure S1.** Age distribution.

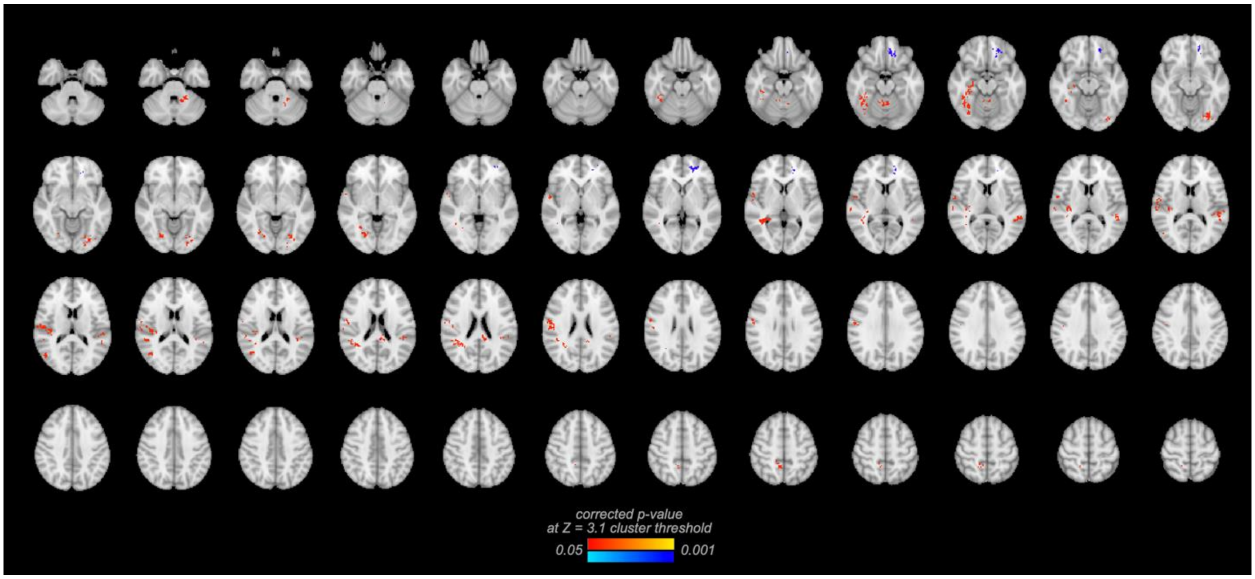


**Figure S2.** The whole-brain map of LC seed-based functional connectivity showing the quadratic effects of age.

*Group-level LC connectivity analysis irrespective of age.*

We performed a group level analysis including the same nuisance regressor (i.e., gender) to see the LC connectivity pattern without age consideration. As a result, we found significant LC connectivity to the cerebellum, brainstem, midbrain, thalamus, and posterior cingulate gyrus. This finding is consistent with previous findings [1-4]. It is noteworthy that the significant brain regions of the group-

level analysis are quite different from that of quadratic model, suggesting that the LC connectivity change differently as a function with age that is not captured by the group-level grand mean analysis.

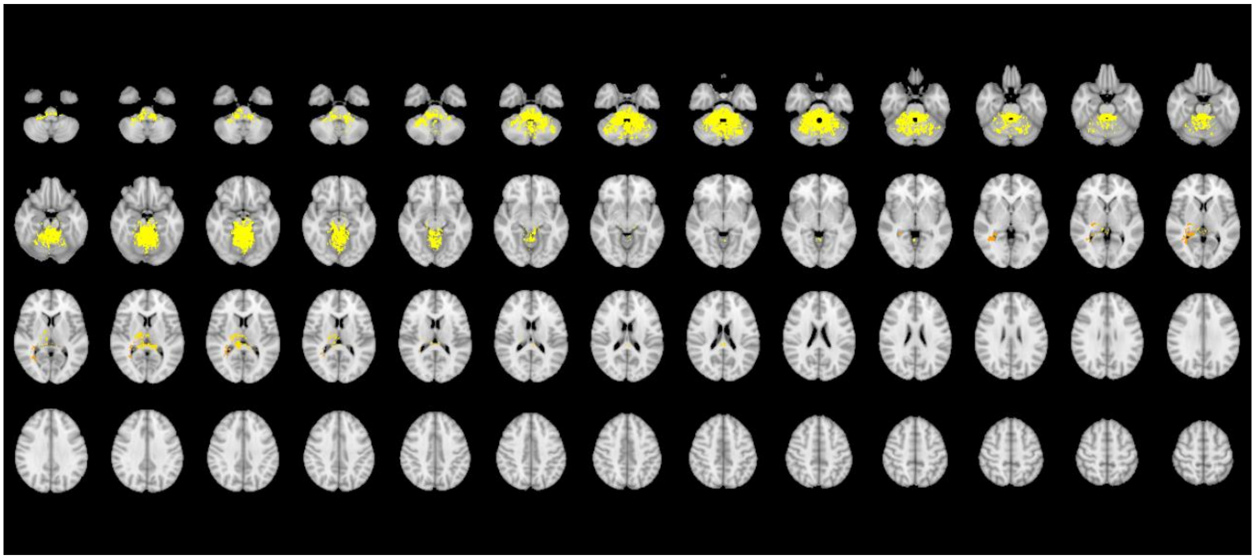

**Figure S3.** Group-level mean LC connectivity without age consideration.

## REFERENCE


1. Zhang, S.; Hu, S.; Chao, H.H.; Li, C.S. Resting-State Functional Connectivity of the Locus Coeruleus in Humans: In Comparison with the Ventral Tegmental Area/Substantia Nigra Pars Compacta and the Effects of Age. *Cereb Cortex* **2016**, *26*, 3413-3427, doi:10.1093/cercor/bhv172.
2. Liebe, T.; Kaufmann, J.; Li, M.; Skalej, M.; Wagner, G.; Walter, M. In vivo anatomical mapping of human locus coeruleus functional connectivity at 3 T MRI. *Human brain mapping* **2020**, *41*, 2136-2151.
3. Aston-Jones, G.; Waterhouse, B. Locus coeruleus: From global projection system to adaptive regulation of behavior. *Brain Res* **2016**, *1645*, 75-78, doi:10.1016/j.brainres.2016.03.001.
4. Breton-Provencher, V.; Drummond, G.T.; Sur, M. Locus Coeruleus Norepinephrine in Learned Behavior: Anatomical Modularity and Spatiotemporal Integration in Targets. *Front Neural Circuits* **2021**, *15*, 638007, doi:10.3389/fncir.2021.638007.
